# The Role of Lipid Biomarkers in Major Depression

**DOI:** 10.3390/healthcare5010005

**Published:** 2017-02-03

**Authors:** Amy Parekh, Demelza Smeeth, Yasmin Milner, Sandrine Thuret

**Affiliations:** Basic and Clinical Neuroscience, Institute of Psychology, Psychiatry and Neuroscience, King’s College London, 125 Coldharbour Lane, London SE5 9NU, UK; amy.parekh@kcl.ac.uk (A.P.); demelza.smeeth@kcl.ac.uk (D.S.); yasmin.milner@kcl.ac.uk (Y.M.)

**Keywords:** lipid biomarkers, major depressive disorder, cholesterol, polyunsaturated fatty acids

## Abstract

In the UK, the lifetime-documented prevalence of major depressive disorder (MDD) is currently 10%. Despite its increasing prevalence and devastating impact on quality of life, the pathophysiological mechanisms underpinning MDD remain to be fully elucidated. Current theories of neurobiological components remain incomplete and protein-centric, rendering pharmacological treatment options suboptimal. In this review, we highlight the pivotal role of lipids in intra- and inter-neuronal functioning, emphasising the potential use of lipids as biomarkers for MDD. The latter has significant implications for improving our understanding of MDD at the cellular and circuit level. There is particular focus on cholesterol (high and low density lipoprotein), omega-3, and omega-6 polyunsaturated fatty acids due to established evidence in the literature of a link between atherosclerotic disease and major depression. We argue that there is significant potential scope for the use of such peripheral biomarkers in the diagnosis, stratification and treatment of MDD.

## 1. Introduction

Major depressive disorder (MDD) is a prevalent psychiatric disorder, now predicted to be the second leading cause of disability by 2020 [1,2]. In the UK, the prevalence of a single lifetime episode of major depression is 6.4% [3]. According to the Diagnostic and Statistical Manual of Mental Disorders IV (DSM-IV), the main symptoms that define the diagnostic criteria for MDD are low mood, anhedonia and anergia, lasting for a period of at least two weeks [4]. The presence of these symptoms severely impairs daily functionality and contributes to loss of productivity [5,6]. MDD is associated with a variable prognosis and chronic course; the median duration of an episode is reported to be 23 weeks, with 25% of individuals proceeding to suffer further episodes in their lifetime [7]. In addition to having reduced quality of life, MDD patients often have a shorter life expectancy than healthy individuals, in part due to an increased lifetime suicide rate [8], but also due to higher death rates associated with comorbid disorders, most notably cardiovascular disease (CVD) [9]. Furthermore, persistent and pervasive low mood leaves sufferers with a significantly impaired quality of life.

In clinical practice, much of the challenge of the diagnosis, management and treatment of MDD lies in the current lack of understanding of the molecular and cellular pathophysiology. Thus, the current situation sees diagnosis and treatment based on various psychiatric rating scales. Whilst these aim to reduce subjectivity and inter-clinician variability, they are arguably inadequate to provide a complete assessment and appropriate treatment plan [10]. In addition to inadequacies in diagnosis, up to 30% of patients do not respond to antidepressant medication and are diagnosed as having treatment-resistant depression [11]. Those that do exhibit a response often only receive a partial reduction in symptoms and a large proportion of the apparent efficacy of antidepressants has been proposed to be due to the placebo effect [12].

One potential avenue for improving the clinical management of MDD is the use of peripheral biomarkers rather than subjective symptom scoring. If effective, measuring peripheral biomarkers could be an objective, cost-effective, time-efficient and non-invasive method of diagnosing and monitoring MDD. Research to date has proposed candidate biomarkers based on various hypotheses, most notably the role of monoamine neurotransmission, immune-inflammation, neuroplasticity and neuroendocrine function [13]. Central to most of these theories is protein dysfunction. However, lipids have a critical role in determining the cellular function of proteins by regulating transport, anchoring and structural support. Furthermore, lipids are fundamental to neuronal function, with numerous roles including the regulation of membrane fluidity and permeability, vesicular formation and transport, neurotransmitter release, cell integrity and plasticity [14,15].

Lipids represent a potential family of peripheral biomarkers that can be utilised for quantitative diagnosis, monitoring treatment response and patient stratification. Their association could also indicate a disease mechanism that is amenable to pharmacological intervention or preventative strategies through dietary supplementation.

## 2. Cholesterol

Cholesterol is one such peripherally detectable lipid, with defects in its metabolism, transport or quantity potentially playing a role in MDD [16]. Of total body cholesterol, 20% resides in the brain, 70% of which is found in myelin-forming oligodendrocytes [17,18]. Despite its abundance, cholesterol levels are tightly regulated due to its requirement for appropriate neurological development and function [19]. Cholesterol is found in plasma membranes where its location and concentration affects membrane fluidity [20]. This in turn impacts the regulation of membrane-bound proteins and ion channels and subsequently synaptic transmission. It is further required for synapse formation, dendrite formation and axonal guidance. A failure in any one of these leads to failed neurotransmission and decreased synaptic plasticity. Aberrations in many of these functions are found to be altered in depression [21].

While a component of the human diet, ingested cholesterol is generally poorly absorbed. Instead, cholesterol is predominantly synthesised in human cells by a complex synthetic pathway [22]. This is a multi-step process controlled by a series of enzymes. Due to the inability of cholesterol to cross the blood–brain-barrier, the majority of brain cholesterol is recycled or synthesised locally, mainly by astrocytes and oligodendrocytes. This process is tightly coupled to the transport of cholesterol. Cholesterol is carried in the circulation by binding to apolipoproteins to form lipoproteins. There are various subtypes of apolipoprotein which form different density lipoproteins, all of which function to control transportation, solubility and stability of cholesterol [23]. Whilst the link between peripheral and central nervous system (CNS) metabolism is unclear, it has been shown that a cholesterol-rich diet and chronic cholesterol supplementation can change the CNS lipid profile of rats [23] and that cholesterol-lowering statins can reduce brain cholesterol synthesis in guinea pigs [24]. Future studies are needed to determine the potentially bidirectional effect between peripheral and CNS cholesterol profile.

### 2.1. Cardiovascular Disease and Major Depressive Disorder

CVD covers a wide range of diseases that involve blood vessels and the heart, and are often found in patients also suffering from MDD. Indeed, when compared to non-depressed individuals, depressed patients have a 2–4-fold increased risk of developing coronary heart disease [25,26]. Further studies have found that suffering from MDD increases the risk of cardiac mortality, sometimes independently of CVD status at baseline [26,27]. Conversely, acute and chronic depressive periods are common following a myocardial infarction and have an adverse effect on patient outcome [28,29,30]. Whilst it is clear there is a relationship between CVD and MDD, the nature of the association as causative or correlative remains to be elucidated. A further question to be answered is whether MDD and CVD are early and late complications of the same underlying pathophysiology respectively.

Various shared pathological mechanisms have been suggested for the increased risk of CVD and cardiac mortality in depressed patients, including altered cholesterol metabolism. Many forms of CVD are due to atherosclerosis, which is initiated by the accumulation of atherogenic lipids, such as the cholesterol-carrying low-density lipoproteins (LDL), in the blood vessel wall [31]. Here LDL becomes oxidised and triggers an immunological cascade starting with adhesion of monocytes and release of cytokines from the injured endothelial wall. Macrophages accumulate and scavenge lipids to form foam cells, while further infiltration of immune cells such as natural killer cells, mast cells and dendritic cells, occurs. Subsequently, the blood vessel endothelium undergoes tissue remodelling and a plaque is formed. In later stages this can lead to narrowing of the lumen, blood vessel rupture or plaque rupture. In addition to the role of LDL, a deficit of high-density lipoproteins (HDL) contributes to atherosclerosis. HDL has anti-inflammatory properties, inhibits oxidation of LDL and can remove cholesterol from foam cells [32].

### 2.2. HDL and LDL in Major Depressive Disorder

In addition to CVD, levels of cholesterol and cholesterol-containing molecules have been linked to MDD. Decreased total serum cholesterol is commonly observed in depressed patients suffering from MDD when compared to healthy controls [33,34,35]. Furthermore, a considerable body of research has demonstrated that MDD patients often exhibit a decrease in high-density lipoprotein, an increase in low-density lipoprotein and an increase in LDL/HDL ratio [21,34,36]. Interestingly, lack of remission from depressive symptoms is also associated with low levels of both total serum cholesterol and LDL cholesterol [37,38]. In addition to the observed association with differing HDL and LDL levels, the levels of constituent apolipoproteins have been associated with MDD [39]. A case-control study revealed that depressed patients had higher circulating levels of LDL and its apolipoprotein B (apoB, constituent of LDL), alongside lower levels of HDL and apolipoprotein A (apoA, constituent of HDL) when compared to healthy controls.

There are studies that do not support these findings and claim a lack of significant association between low serum cholesterol and depression in elderly cohorts [40,41]. Whilst it is invaluable to examine lipid levels in different age groups, without a fuller understanding of the mechanisms underlying major depression, it is difficult to say whether patients over the age of 65 share the same pathological processes as younger patients. Some studies have provided mixed results with increased very low density lipoprotein (VLDL) serum levels, but no change in total cholesterol or HDL levels [42], while others report increased total cholesterol in depressed groups [21].

Although many studies indicate a potential relationship, there remain inconsistencies in this link between cholesterol species and major depression, and the latter with CVD. A meta-analysis aiming to evaluate associations among total, high- and low-density lipoprotein and depression concluded that total cholesterol and depression were inversely related, while increased HDL levels were related to increased depression, especially in women [43]. Furthermore, the strongest association was within medically naïve samples, presenting the notion that patients should be monitored. A more recent meta-analysis concluded that circulating LDL levels were inversely associated with depression [44]. Wider studies across different cohorts must be conducted to establish the nature of the link between cholesterol and depression and respond to the discrepancies in results, in order for cholesterol to be useful in diagnosis. Additionally, the potential relationship between LDL levels and depressive symptoms could be confounded by factors such as body mass index (BMI) [45]. Increased LDL levels are associated with increased BMI, which itself has been associated with depressive symptoms. Many studies have found that increased BMI is associated with an increased risk of mood disorders such as MDD [46,47]. A further study found an inverse association between BMI and MDD but no association with other measures of obesity, indicating that increased muscle mass rather than obesity may decrease depression risk [48].

### 2.3. Cholesterol and Other Psychiatric Disorders

Suicide or suicidal ideation is a rare but devastating symptom of MDD. Aberrant cholesterol in patients with MDD is associated with increased suicidality [49]. There is a wealth of recent data that highlights the significant correlation between low circulating total cholesterol and suicidality [16,50,51,52,53,54,55,56,57]. Specifically, depleted serum cholesterol levels were associated with suicidal ideation, history of suicide attempts, a more severe and violent method of suicide, and an increased likelihood of having a first-degree relative who completed suicide in various inpatient and outpatient analyses [58,59,60,61,62,63]. Conversely, no link was found between suicide attempt and any blood lipid value in patients with bipolar disorder [64]. This indicates that low cholesterol levels could be associated with particularly severe cases of MDD or indicate the likelihood of suicide attempt. The use of cholesterol biomarkers in this case aid with identifying particular groups of depressed patients which need additional monitoring and support.

Whilst understanding the mechanisms underpinning MDD would be a pivotal advancement, the relationship between cholesterol and other psychiatric disturbances may provide insight into this topic. Generalised anxiety disorder (GAD) is associated with elevated total serum cholesterol and LDL, and decreased HDL [65,66,67]. That is not to say that these two disorders are opposite ends of a single spectrum, but they certainly present some opposing features in this instance. Interestingly, patients with comorbid MDD and GAD present an exacerbated lipid profile differing from each disease alone [65,66,67].

Other studies have found elevated serum cholesterol levels in patients with other anxiety disorders such as panic disorder, obsessive compulsive disorder and phobia [66,68,69], with the increase in LDL and VLDL levels correlating with symptom severity [70]. Furthermore, comorbidity of these anxiety disorders with MDD produces further differing lipid profiles [69,71]. One might infer that the correct levels of cholesterol are important in preventing both depressive and anxious disorders and that there is an optimum range within which they should lie. Furthermore, it indicates the need to consider the presence of comorbid disorders when considering the use of cholesterol as a biomarker of MDD.

### 2.4. Mechanism of Action of Cholesterol

The proposed molecular mechanism for the inverse relationship between serum cholesterol levels and suicidality is via effects on the serotonin (5-HT) system, currently one of the main targets of antidepressant therapy [72]. Low cholesterol content in cell membranes is associated with decreased density of 5-HT receptors measured in in vitro mouse analysis [72,73,74]. Sun et al. exposed rats to chronic mild stress (CMS) for 28 days and showed significantly reduced total cholesterol level in the medial prefrontal cortex (mPFC) [74]. Chronic dietary supplementation reversed the behaviours induced by CMS. Furthermore, injection of a 5-HT1A antagonist into the mPFC blocked the treatment effects of cholesterol supplementation [74]. This suggests that cholesterol, particularly in the prefrontal cortex, influences the sensitivity of the 5-HT1A receptor in the pathology and treatment of depression. However, the serotonergic explanation of depression is not sufficient to fully explain the development of depression, as evidenced by large proportion of patients that do not respond to antidepressants which target the serotoninergic system [11,75,76].

Another potential mechanism is via an altered inflammatory profile. As discussed earlier, high LDL and low HDL contribute substantially to immunological upregulation in the development of atherosclerosis. Specifically, high concentrations of cholesterol increase the release of interleukin 6 (IL-6) and tumour necrosis factor alpha (TNFα) in hypercholesteraemic rabbits and from cholesterol-treated macrophages in vitro [77,78]. IL-6 and TNFα levels are often increased in depressed patients and decreased by successful antidepressant therapy [79,80]. Furthermore, oxidised LDL treatment in rabbits and human aortic smooth muscle cells led to increased production of interleukin 1β (IL-1β) [81], a cytokine which has also been found to be upregulated in depressed patients [82].

### 2.5. Targeting Cholesterol Pathways as Antidepressant Therapy

Despite some evidence to suggest a role for cholesterol within the pathophysiology of depression, it remains to be seen whether it could provide a new target for therapeutic intervention. It has been shown that individuals suffering from mood disorders with a pathologically decreased serum cholesterol level displayed a concomitant reduction in symptom severity and increase in serum cholesterol following intervention with psychological and/or pharmacological intervention [83]. This indicates that changes in peripheral cholesterol are found alongside lessening of symptoms and indicates the potential use of cholesterol biomarkers in monitoring treatment.

The use of cholesterol as a dietary supplement within MDD therapy is unlikely to gain credit. Certainly, the effects of aggressive cholesterol therapy yield positive results with regard to CVD [84]. However, the viability of such treatment through diet alone is questionable. Furthermore, without a clear relationship between depression and cholesterol levels, it is difficult to recommend any treatment that targets cholesterol levels.

It may be possible to target the cholesterol pathway via other means, for example the use of cholesterol-reducing statins. A double-blind trial using lovastatin as an adjunct to fluoxetine showed a more significant reduction of depression on the Hamilton Depression Scale in the treatment group when compared to the control [85]. A further double-blind trial showed that adjuvant atorvastatin with citalopram produced a more pronounced antidepressant effect than citalopram alone, supporting the idea that statins can help reduce depressive symptoms [86]. Furthermore, statin use in general is associated with a 32% lower risk of developing depression [87]. Statins are known to reduce cholesterol synthesis and increase uptake of peripheral LDL, which supports the role of increased LDL in maintaining the course of MDD. Additionally, it is important to establish whether HDL supplementation carries any benefit, as little research has been conducted in this area.

Current therapies for treatment-resistant depression include electroconvulsive therapy (ECT) and exercise [88]. ECT has been shown to increase total cholesterol levels [89], while exercise decreases LDL and increases HDL levels, supporting the notion that cholesterol could be involved in MDD and can be rectified.

## 3. Polyunsaturated Fatty Acids (PUFAs)

**Figure 1:**
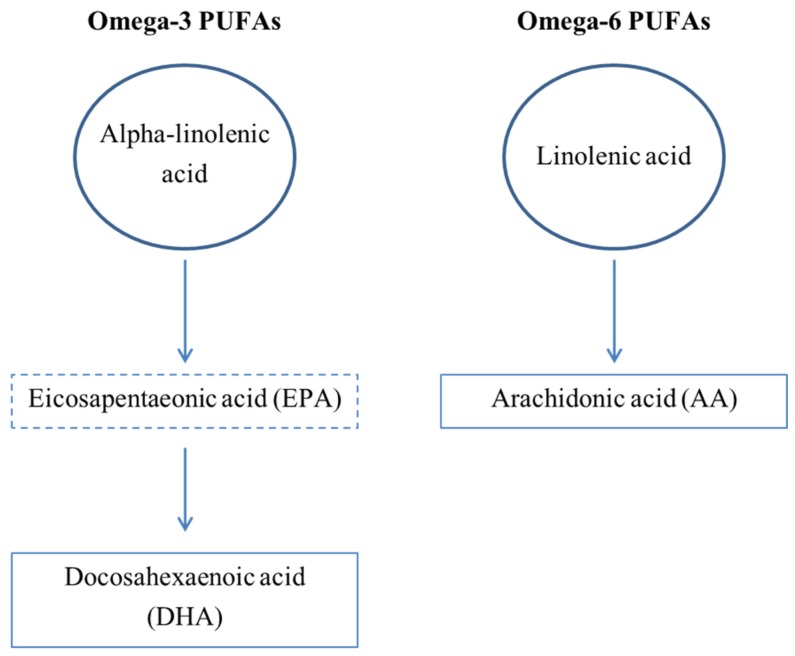
Subgroups of PUFAs. There are many members of the polyunsaturated fatty acid (PUFA) family; the most relevant to this discussion are shown in the above diagram. Arachidonic acid (C20:4) is derived from linolenic acid/omega-6 (C18:2). Docosahexaenoic acid (C22) derives from α-linolenic acid/omega-3 (C18:3) via the precursor eicosapentaeonic acid (C20:5).

Like cholesterol, polyunsaturated fatty acids (PUFAs) are detectable in the peripheral circulation and have been linked to MDD. Phospholipids which form membranes are comprised of phosphate “heads”, glycerol and fatty acid “tails”. These PUFAs are either derived from linolenic acid (omega-6) or alpha-linolenic acid (omega-3; Figure 1). The most common omega-6 is arachidonic acid, whilst the most common omega-3 is docosahexaenoic acid (DHA), followed by its precursor eicosapentaenoic acid (EPA) [90]. DHA alone comprises 15%–20% of human brain lipid and reduction in this group has strong association to depression due to dysregulation of inflammatory responses, decreased antioxidant capacity and disordered neurotransmission [91]. DHA is the most abundant omega-3 fatty acid in the mammalian CNS, particularly during early developmental stages [92]. It is absent from foods of plant origin, with the richest dietary source being fatty fish [93]. Omega-6 PUFAs are generally pro-inflammatory, whose major detrimental effect is thought to be due to competitive inhibition of omega 3 PUFAs, indicating a protective effect of omega-3 PUFAs [90]. Both omega-3 and omega-6 PUFAs are essential compounds, meaning they can only be derived from diet (omega-6 is largely derived from plant oils, whilst omega-3 predominately comes from fish oils). That, coupled with the proof that differentiation and functioning of cultured brain cells requires alpha-linolenic acid, omega-3 and omega-6 PUFAs, makes the link between brain function and diet clearer [94].

### 3.1. Absolute PUFA Levels and MDD

Deficits in omega-3 PUFA levels, both free in the circulation and residing in erythrocyte membranes, have been observed in depressed patients when compared to non-depressed controls [95,96,97,98]. Furthermore, the severity of depressive symptoms is associated with a more pronounced decrease in omega-3 PUFAs [99]. The idea that low omega-3 PUFAs levels is linked to depression is supported by the observation that lower rates of depression are found in countries which have a higher fish consumption, a major source of dietary omega-3 PUFAs [100,101]. Furthermore, a wealth of studies has found an inverse relationship between consumption of fish and incidence of depression in various populations [102,103,104,105,106,107].

### 3.2. Ratios of PUFA Sub-Types and MDD

Humans are originally thought to have evolved with a 1:1 ratio of omega-6:omega-3 in their diet. Over the past 100 years the consumption of omega-3 PUFAs has declined, alongside an increased consumption of omega-6 producing dietary fatty acid ratios of 15/16:1 [108,109,110]. This coincides with the recent increase in depression diagnosis, which indicates that this dietary imbalance may play a role [111]. This suggests that ratios of PUFAs may be equally if not more important than absolute levels in the development of MDD.

This hypothesis is supported by multiple studies which have observed higher omega-6:omega-3 PUFA ratios in depressed patients [95,112,113,114]. Indeed, it has been observed that more severe cases of depression are associated with increased omega-6:omega-3 PUFA ratios, in both plasma and erythrocytes [110,112]. Likewise, a longitudinal study also showed that an increased omega-6:omega-3 ratio at baseline was associated with a worsening of depressive symptoms over time, indicating a potential causal relationship [115]. There are some studies which have not replicated these findings [116,117], but the majority of studies support the idea that a higher omega-6:omega-3 PUFA ratio is associated with depression [118]. It is clear that it is not only the absolute concentration of each PUFA species which is important, but rather their relative ratios.

### 3.3. Mechanism of Action of Polunsaturated Fatty Acids

The many sites of omega-3/omega-6 PUFA action are beginning to be observed in depressed patients. Certainly, altered membrane fluidity has been postulated in MDD, demonstrated by increased membrane fluidity with omega-3 PUFA supplementation [119,120]. Further roles affected by omega-3 PUFAs are neuronal development, inflammation and neurotransmission: a reduction in serotonergic neurotransmission parallels decreased dietary DHA in piglets and is observed in depressed patients [121,122,123,124,125]. This is in conjunction to an alteration of the sensitivity of the 5-HT receptor in deranged cholesterol levels [63,126]. Although the underlying mechanism has not yet been elucidated, there certainly appears to be a reduction in serotonergic neurotransmission in MDD patients, further supported by the fact that the most effective antidepressants, such as fluoxetine, are specific serotonin reuptake inhibitors (SSRIs). Research has accepted that certain neurotransmitters, notably endocannabinoids, are synthesised from lipids [127]. Scope is therefore left to establish whether there is a direct effect of lipid dysfunction on neurotransmitter synthesis, storage or release.

Omega-6 PUFAs are generally considered to be pro-inflammatory, whilst omega-3 PUFAs are thought to be anti-inflammatory. This inflammatory balance is based on the derivation of eicosanoids, small signalling molecules that act as messengers in the CNS and peripherally to govern inflammation. During the inflammatory cascade, omega-6 prostaglandin and leukotriene eicosanoids are synthesised from arachidonic acid. Those derived from omega-3 PUFAs are less inflammatory or even anti-inflammatory [127]. This action of omega-3 PUFAs is by countering the effects of arachidonic acid-derived eicosanoids, either via displacement, competitive inhibition or counteraction [128]. These essential nutrients mean the amount and balance of omega-3/omega-6 PUFAs is determined by a person’s dietary intake, which therefore determines eicosanoid-controlled functions. Given the role of eicosanoids in inflammation, their derivation from PUFAs and the link between inflammation and MDD, it is perhaps unsurprising that decreased omega-3 PUFA levels and increased omega-6 PUFA levels are observed in patients with major depression. Furthermore, DHA-deficient rats show a marked increase in IL-6 and TNFα [13,129,130], which were reversible upon normalisation of DHA levels [13]. IL-6 and TNFα levels, as previously described, are often elevated in depressed patients [79,80].

### 3.4. Supplementation of Omega-3 PUFAs as A Potential Antidepressant Therapy

Administration of DHA and EPA have been the focus of numerous intervention studies in humans aiming to reduce the symptoms of MDD via omega-3 PUFA supplementation and have yielded promising but mixed results. Lee et al. [131] showed substantial evidence supporting their use as effective therapy. Patients given esterified EPA alongside their unchanged pharmaceutical regimen showed a significant reduction in their depressive symptoms. Peet and Horrobin carried out a double blind dose-ranging study on the effects of EPA in patients with ongoing MDD. This showed that a low daily dose of EPA improved depressive symptoms, while higher doses had no therapeutic effect [132].

Meta-analyses of the literature show that MDD patients have lower omega-3 PUFA levels than control groups and that the supplementation of omega-3 shows significant benefit in symptoms of depression [133,134]. A meta-analysis of 15 double-blinded, placebo-controlled trials comprising 916 participants concluded that omega-3 PUFAs were an effective and safe antidepressant therapy in MDD and related mood disorders [135]. This meta-analysis included studies trialling omega-3 PUFA augmentation of current antidepressants therapies. Furthermore, this analysis concluded that EPA was a more effective anti-depressant than DHA, and omega-3 PUFA therapy is most successful when the formulation contains greater proportion of EPA than DHA. A more recent meta-analysis of randomised controlled trials covering omega-3 PUFA treatment of MDD suggested that this intervention produces a non-significant trend for efficacy in trials with poor methodological quality, short durations and patients with more severe depression at baseline, while 13 trials showed no significant benefit [136]. This data suggests that overall omega-3 PUFA supplementation may represent a potential anti-depressant therapy, at least in some cohorts. Given that that the modern Western diet is often deficient in omega-3 PUFAs, it may be that the treatment simply rectifies this imbalance. Currently, there is no research looking at whether reducing omega-6 PUFA intake has any benefit.

Studies in humans have also shown the possible benefit of DHA dietary supplementation for reducing stress-related behaviours such as aggression [137,138,139]. Whilst these studies are limited in their size and absence of mental health patients, taken together they further support the notion of omega-3 PUFA supplementation in order to alter behaviour. It is worth noting that some research has centred on populations with a naturally very high fish intake [100,102] and trials examining omega-3 PUFA supplementation in MDD have produced inconclusive results.

Thus far, human studies have yielded promising results. However, further reproducible trials with larger cohorts are required to assess the significance of these findings. However, numerous animal studies have been performed that indicate omega-3 PUFAs may be a promising MDD therapy in certain cases. Rats and mice fed a diet deficient in omega-3 PUFAs exhibit depressive-like behaviour and deficits in learning, which can be partially rectified by supplementing omega-3 PUFAs back in to the diet [140,141,142]. This indicates that omega-3 PUFA supplementation can have partial anti-depressant effects if a deficiency already exists. Furthermore, these studies found that while a deficient diet reduced omega-3 PUFA content in the brain, supplementation rectified this change [140,141,142]. These papers raise questions as to whether the importance of brain lipid state is laid out during suckling and development or whether there is the potential for alteration during adulthood.

### 3.5. Role of Enzymes in Fatty Acid Metabolism

With regard to both PUFAs and cholesterol, it is unknown where in the pathway the dysfunction occurs. It is possible and has been suggested that there is alteration in the enzymes responsible for neuronal fatty acid metabolism such as long-chain acyl-CoA synthetase (LACS-2) and those implicated in endocannabinoid regulation, such as fatty acid amide hydrolase (FAAH). Long-chain acyl-CoA synthetase is a crucial enzyme in fatty acid metabolism in the brain [143]. Increased LACS-2 mRNA expression has been observed in rats exposed to the learned helplessness paradigm, an animal model of depression. This trend was observed in 84% of brain regions examined, including the frontal cortex [144]. Furthermore, FAAH levels are higher in the frontal cortex and hippocampus of rats displaying depressive-like symptoms [145]. In the future, the role of fatty acid metabolism in relation to circuit functioning in MDD with respect to other signalling systems beyond endocannabindoids will require further analyses, as currently this is limited. Combining analysis focused on enzyme activity with further profiling of lipid profile dysregulation in MDD has great potential to yield specific targets for therapeutic interventions in the future.

## 4. Other Lipids Biomarkers and MDD

Although this review has focussed on the potential roles of cholesterol and polyunsaturated fatty acids and biomarkers of depression, there are many other lipid species which have been linked to MDD, including glycerolipids, sphingolipids, glycerophospholipids and triglycerides [23]. This is not to say that they are less important, rather there is a smaller pool of evidence supporting their role in depression. Furthermore, while the serum concentrations of single lipid species have been linked to MDD, it may be more useful to consider the entire lipid profile composed of several lipid species of interest, potentially alongside the more traditional peptide and metabolite biomarkers [146].

In addition to lipid-based molecules, there are many peptide-based biomarkers which are associated with the intake, transport and metabolism of lipid species. The peptides leptin and neuropeptide Y are both involved in appetite control and energy homeostasis [147,148] and have links to stress and depression [149,150,151]. The use of these peptide biomarkers involved in lipid homeostasis could complement the monitoring of lipid biomarkers in depression.

## 5. Discussion

**Figure 2:**
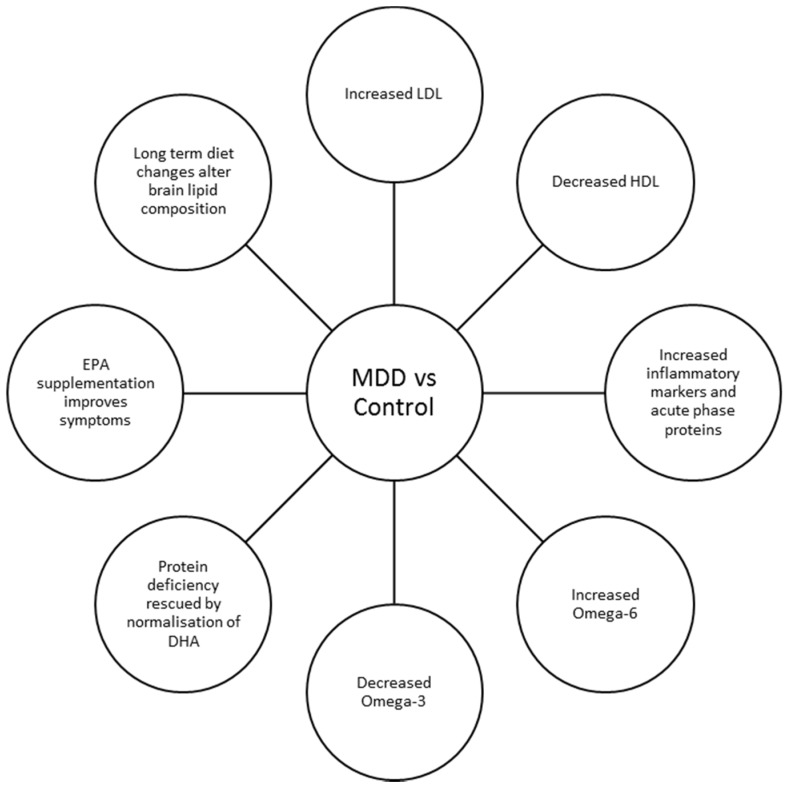
The role of lipids in major depression disorders (MDD). The various potential roles for lipids in the cellular pathophysiology underpinning MDD are shown in this diagram. The main findings when comparing MDD patient with controls is an altered lipid profile, most specifically that low-density lipoproteins (LDL) and omega-6 levels are raised, where high-density lipoproteins (HDL) and omega-3 levels are decreased. EPA: eicosapentaeonic acid; DHA: docosahexaenoic acid.

Recent research has highlighted the potential role of monitoring peripheral PUFAs and cholesterol in the prediction, stratification and management of MDD (Figure 2). However, preliminary conclusions such as the reciprocal decrease in HDL and omega-3 PUFAs and the increase in LDL and omega-6 PUFAs remain to be further analysed to sufficiently understand the relationship before using these lipids as biomarkers of depression. First and foremost, it will be pertinent to clarify whether such changes in lipid homeostasis are the driving force of MDD pathophysiology, or whether they are a consequence of the disease. Certainly, if the effect is causal it is far more likely that an alteration in the intake or metabolism of these lipids drives the pathophysiology of depression than it is possible that depression drives the reduction in lipids. Further, it is unknown whether the changes result from altered intake or metabolism. This leaves scope to determine the roles of enzymes in lipid synthesis with relation to mental illness. Furthermore, it may be that a relationship is only seen in a subset of patients. This leaves the possibility of defining subtypes of depression based on their lipid biomarker profile.

Inflammatory processes are known to be crucial and further research into PUFAs and cholesterol may show a complex relationship between these factors in the pathology of MDD. Indeed, it has been shown that increased HDL and omega-3 PUFAs could protect against depression-mediated inflammation [74,152]. The precise mechanics of inflammatory processes continues to place protein dysfunction as a key aspect of MDD pathophysiology, but the role of lipids is proving to be just as important, especially since protein function at the molecular level is largely determined by lipid function.

Whilst modern antidepressant medications can be effective, prescribed regimens result in only a partial improvement in 38% of patients clinically [62]. There is no doubt that there is need to identify novel therapeutic targets and devise more efficacious treatments for MDD. Two common pathways indirectly altered by current pharmacological treatments for MDD are neuro-protection and anti-inflammation [153]. These two processes are also targets of omega-3 PUFAs, making the potential role of PUFAs in MDD promising. Populations with a high fish intake demonstrate lower prevalence of MDD, and MDD patients have lower levels of omega-3 PUFAs [102], suggesting that this family of molecules can exert a protective effect, although the precise molecular mechanism through which this arises remains to be determined. Certainly, not all studies involving supplementation of omega-3 PUFAs showed a significant improvement of symptoms, but the heterogeneity of MDD pathophysiology may require multiple, simultaneous supplementations. Certain patients respond better to certain medications, indicating the complexity of MDD in terms of the contributing physical, psychological and social factors to diagnosis, prognosis and treatment response [154]. Furthermore, there could be scope in the future to sub-categorise within MDD such that omega-3 PUFA-sensitive depression could be defined and targeted more specifically, with the hope of more efficacious outcomes. If this is the case, there is the advantage that omega-3 supplementation confers no known side effects, is safe and cost-effective. In this sense, the identification of patients who may respond to this adjunctive therapy would prove wholly beneficial. That said, augmenting current therapies with omega-3 may not be feasible given the unknown and potentially large quantities required.

## 6. Conclusions

The question remains as to whether there is sufficient evidence to apply the use of lipids in the diagnosis and treatment of MDD. Certainly, it would be pertinent to perform further analyses in future, especially given the ease and low cost with which supplementation can be provided and peripheral blood markers can be measured. The variety in response to omega-3 PUFA supplementation suggests that with larger cohort studies in future, patients could be stratified according to symptoms and MDD severity, fostering a more specific and targeted approach to diagnosis and treatment. This leaves scope for larger trials to assess whether these, and other, lipid biomarkers are as important as they seem from research studies to date.
